# Overcoming bubble formation in polydimethylsiloxane-made PCR chips: mechanism and elimination with a high-pressure liquid seal

**DOI:** 10.1038/s41378-024-00725-1

**Published:** 2024-09-27

**Authors:** Shiyuan Gao, Tiegang Xu, Lei Wu, Xiaoyue Zhu, Xuefeng Wang, Xiaohong Jian, Xinxin Li

**Affiliations:** 1 State Key Laboratory of Transducer Technology, Shanghai Institute of Microsystem and Information Technology, Chinese Academy of Sciences, Shanghai, 200050 China; 2 College of Materials Science and Opto-Electronic Technology, University of Chinese Academy of Sciences, Beijing, 100049 China; 3 School of Information Science and Technology, ShanghaiTech University, Shanghai, 201210 China; 4 Fujian Provincial Key Laboratory of Haixia Applied Plant Systems Biology, Metabolomics Center, Haixia Institute of Science and Technology, School of Future Technology, Fujian Agriculture and Forestry University, Fuzhou, 350002 China; 5 School of Biological Engineering, Sichuan University of Science and Engineering, Yibin, 644000 China

**Keywords:** Physics, Chemistry, Engineering

## Abstract

The thermal expansion of gas and the air permeability of polydimethylsiloxane (PDMS) were previously thought to be the main causes of bubbles and water loss during polymerase chain reaction (PCR), resulting in a very complex chip design and operation. Here, by calculating and characterizing bubble formation, we discovered that water vapor is the main cause of bubbling. During PCR, heat increases the volume of the bubble by a factor of only ~0.2 in the absence of water vapor but by a factor of ~6.4 in the presence of water vapor. In addition, the phenomenon of “respiration” due to the repeated evaporation and condensation of water vapor accelerates the expansion of bubbles and the loss of water. A water seal above 109 kPa can effectively prevent bubbles in a bare PDMS chip with a simple structure, which is significant for the wide application of PDMS chips.

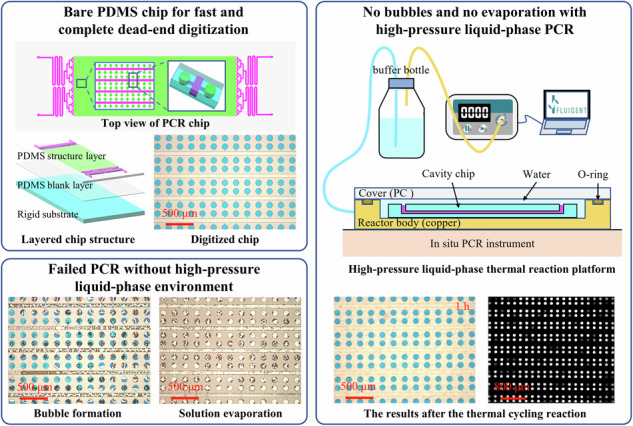

## Introduction

For many years, quantitative PCR technology has been widely used in areas such as cancer screening, virus detection, and prenatal diagnosis due to its ability to accurately quantify nucleic acids^1,2^. With the development of microfluidic and microfabrication technologies, microfluidic chips have become increasingly popular for PCR technology because of their small size, multiple reaction units, and fast reaction speed^3–6^.

Various materials, including silicon^7^, glass^8^, and polymers^9^, have been used to fabricate PCR chips. Because of their ease of manufacture, commercial digital PCR (dPCR) chips are typically made of hard plastics, such as polymethyl methacrylate or polycarbonate (PC). However, due to the difficulty of air passing through hard plastic, air sometimes becomes trapped in the chip’s chamber, resulting in inaccurate sample volumes. In contrast, PDMS allows air to pass through easily^10^, enabling complete dead-end filling and rapid sample loading in just 120 s^11^. PDMS also offers the advantage of fabricating microstructures with higher precision, even at the nanometer scale^12^. In addition, PDMS is biocompatible, cost-effective, easy to fabricate, and optically transparent, making it an ideal material for the fabrication of dPCR chips^13,14^.

However, the formation of bubbles and sample moisture loss in PDMS chips during the thermal reaction process are two significant issues^15^. Rapidly formed bubbles in the chambers can displace the solution, causing a loss of volume in the PCR chamber and potential cross-contamination between different reaction units. Excessive water loss during PCR can lead to changes in ion concentration, resulting in the deactivation of polymerase and the inability to amplify nucleic acids^16,17^.

For decades, researchers have extensively studied the mechanisms of bubble formation and sought methods to prevent their occurrence^18–22^. Early studies suggested that gas residues in channel structures, chip defects, and bonding gaps could lead to bubble formation due to air expansion caused by heating. Strategies such as optimizing channel structures and fluid sealing have been employed to reduce bubbles^23,24^. Chen et al. proposed that the surface wettability and permeability of PDMS are crucial factors in bubble formation, and surface hydrophilic treatment was used to prevent external air from entering the reaction chamber^25^. Luke P. Lee et al. proposed that bubbles formed due to the absorption of water by porous PDMS. They suggested reducing bubbles by incorporating circumferential degassing channels and preventing water loss with a top layer of polyethylene (PE)^26^. In addition, other methods have been employed, such as valves^27^, thermally curable oils^28^, pressurized hydration lines^29^, and vapor barriers^30,31^. However, these methods result in complex chamber structures, requiring not only PDMS and rigid support plates but also additional sealing materials such as parylene, poly(vinyl alcohol), Teflon, and PE. Cumbersome operations, such as sealing inlet and outlet ports before PCR, and external tubing and pumping operations during the thermal reaction process, were also necessary.

In a previous study, we proposed that water vapor, rather than air expansion, may be the primary cause of bubble formation^32^. In this study, bubble formation experiments and theoretical analysis showed that the saturation vapor pressure of water vapor was the key factor in bubble formation in PDMS-made PCR chips and should be given priority in PCR chip design. Based on this mechanism, we designed a simple-to-operate, high-pressure liquid reaction system that completely prevented the occurrence of bubbles in PDMS chips during thermal reactions and significantly reduced PCR solution evaporation.

## Results and discussion

### Water vapor is the primary component of bubbles in PDMS chips

PDMS is a mesoporous material that contains air at a volume of 0.11 cm^3^ (standard temperature and pressure, STP)/cm^3^, and gases can pass through the material^33^. Due to the need for evaporation equilibrium, water vapor can enter the PDMS, causing the gas volume to expand and overflow from the PDMS nanopores, resulting in the formation of bubbles on the surface of the PDMS.

**Fig. 1:**
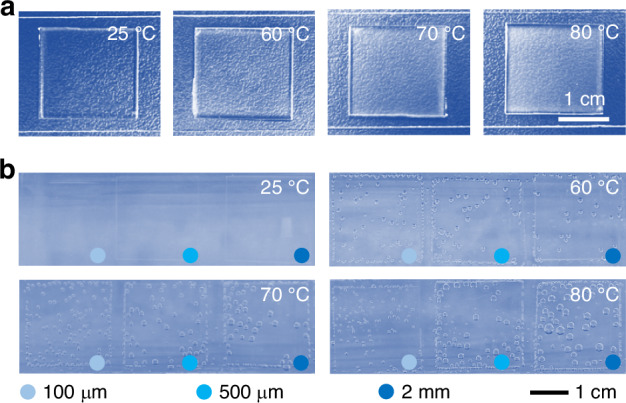
Water vapor penetrated the interior of PDMS and formed bubbles on its surface. **a** Photographs of PDMS blocks at room temperature after being soaked in water at different temperatures for half an hour. The same thickness (2 mm) was used. **b** Isothermal expansion of bubbles. At higher temperatures, bubbles were generated when preheated PDMS blocks were soaked in water at the same temperature for 20 min

To investigate the influence of water vapor on bubble formation, experiments were conducted to demonstrate that water vapor can indeed penetrate PDMS (Fig. 1a). After soaking in water at 25 °C, 60 °C, 70 °C, and 80 °C for 30 min, 2-mm-thick PDMS blocks were exposed to room temperature (25 °C). The PDMS blocks at higher temperatures became cloudy, with the level of cloudiness increasing with temperature. The clouds gradually cleared within 20 min in a room at 40% humidity. The cloudiness in the PDMS was caused by water vapor condensing into small droplets inside the PDMS when it was removed from the water bath and cooled. Due to the dry surroundings, the small droplets in the PDMS reverted to a gaseous state and diffused away, resulting in the PDMS returning to transparency. This finding confirmed that water vapor can enter the PDMS, and higher soaking temperatures lead to more water vapor inside the PDMS, as indicated by the decreased transparency.

Even without thermal expansion, water vapor can still enter the PDMS and form bubbles on the surface of the PDMS in an isothermal expansion experiment (IEE) due to water vapor (Fig. 1b). To eliminate the bubbles caused by thermal expansion, the preheated PDMS blocks were soaked in water at the same temperature. In the IEE, the bottom surface of the PDMS block was bonded to a glass slide, and the gas inside the PDMS could only escape from the top surface of the PDMS, which was surrounded by water, to form bubbles. This process allowed us to compare and analyze the total volume of the bubbles more clearly. The IEE results showed that while no bubbles were observed on the PDMS surface at 25 °C, bubbles were clearly visible at higher temperatures, such as 60 °C, 70 °C, and 80 °C. Furthermore, bubble formation increased with increasing temperature. At 60 °C, very small bubbles appeared on the PDMS surface, whereas at 80 °C, the bubbles were much larger in volume. In the IEE, where gas expansion was absent, thermal expansion was not a necessary condition for bubble formation in the PDMS chips. The vaporization of water alone was sufficient to generate bubbles.

To determine whether the air inside the porous PDMS or the residual air on the PDMS surface played a critical role in bubble formation in the IEE, an experiment was conducted with PDMS of the same size but different thicknesses. The results showed that the volume of bubbles that formed on the surface of the 2-mm-thick PDMS was much larger than that on the 100-μm-thick PDMS (Fig. 1b). The difference in surface area as the PDMS membrane thickness varies is negligible, but volume significantly differs. Therefore, the total amount of air stored inside the porous PDMS is the key factor influencing the formation of bubbles.

In conclusion, in the IEE, water vapor diffused into the dry air stored in the porous PDMS when it was soaked in water at the same temperature, causing an increase in the total gas volume, which overflowed from the porous PDMS and formed bubbles on the PDMS surface. The dry air stored in the PDMS was the source of bubble formation. Thicker PDMS layers stored more dry air, allowing it to accommodate more water vapor, resulting in larger bubbles. Structural features, such as protrusions and defects in the microfluidic channel of the chip, are not necessary conditions for bubble formation. Even a smooth PDMS block without any structures can generate bubbles.

### Extreme value calculation of bubble volume

According to the IEE, water vapor can generate bubbles on the surface of PDMS even without the heating process, which suggests that the impact of water vapor on bubble formation cannot be overlooked. The following is a theoretical analysis and calculation to determine the maximum potential impact of water vapor in the PCR process.

In PCR amplification reactions, the initial temperature of the PDMS chip is typically the same as the room temperature, which is ~25 °C (298.15 K). The chip can reach a maximum temperature of 95 °C (368.15 K) during the process. Therefore, the temperature range for the analysis was 25 to 95 °C.

The IEE without thermal expansion was analyzed first. In the process of water vapor equilibrium, drier air allows for more water vapor absorption. To calculate the maximum increase in bubble volume, we assumed that the gas inside the PDMS was initially composed entirely of dry air. Then, water vapor enters dry air, increasing the partial pressure of water vapor, which can reach the saturation vapor pressure at a given temperature. At this point, the total pressure remains at standard atmospheric pressure (1 atm., 101.325 kPa), while the partial pressure of dry air decreases. The relationship between the pressures of different gases can be described using Dalton’s law of partial pressures:

1 $${p}_{A}=p-{p}_{WV}$$

where *p*_*A*_ is the pressure of air, *p* is the total pressure, and *p*_*WV*_ is the vapor pressure of water. The change in gas volume due to a change in pressure obeys Boyle’s law:

2 $$p{V}_{dry}={p}_{A}{V}_{saturated}$$

where *V*_*dry*_ is the initial volume of dry air and *V*_*saturated*_ is the volume of saturated water vapor. Based on Eqs. (1) and (2), the relative volume increase of water vapor (*ΔV*_*water*_/*V*_*dry*_) in the IEE is given by the following:

3 $$\frac{\Delta {V}_{water}}{{V}_{dry}}=\frac{{V}_{saturated}-{V}_{dry}}{{V}_{dry}}=\frac{{p}_{WV}}{p-{p}_{WV}}$$

**Fig. 2:**
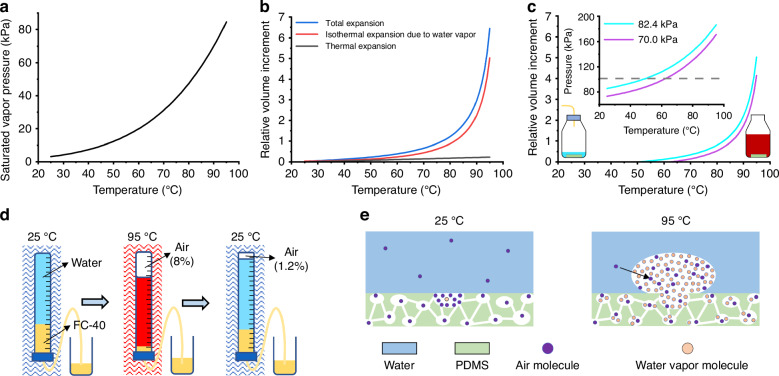
The impact of water vapor on bubble formation during PCR (temperature: 25–95 °C, pressure: 101.325 kPa). **a** Saturation vapor pressure of water. **b** Thermal expansion only causes a slight increase in gas volume (black line), while the equilibrium effect of water vapor leads to a larger volume of isothermal expansion (red line). The synergistic effect of thermal expansion and isothermal expansion of water vapor results in the maximum increase in bubble volume (blue line). **c** Calculated bubble volume generated from degassed porous PDMS during PCR. The inset shows the total pressure of air and water vapor if the volume is constant. **d** The volume of saturated water vapor formed by the air dissolved from the water. Rapid cooling reduced the volume from 8% to 1.2%, indicating that the main component of these bubbles at 95 °C is water vapor. **e** Possible mechanisms for bubble formation involving the equilibrium of water vapor and the overflow of air in the PDMS and PCR solutions

According to Eq. (3), the saturation vapor pressure curve of water within the temperature range of PCR is shown in Fig. 2a. The maximum increase in bubble volume caused by water vapor, *ΔV*_*water*_/*V*_*dry*_, is represented by the red line in Fig. 2b. At 25 °C, when *p* is 101.325 kPa and *p*_*WV*_ is 3.169 kPa, water vapor can increase the volume of dry air by a maximum of 0.03 times. However, when the *p*_*WV*_ is 84.529 kPa at 95 °C^34^, water vapor can maximize the increase in dry air volume by 5.03 times.

The impact of the thermal expansion of gas needs to be considered in the PCR process. The deformation of the PDMS was neglected because the thermal expansion coefficient of the PDMS [i.e., 3.1 × 10^−4^ (1/K)] was 10 times less than that of air [3.4 × 10^−3^ (1/K)]^24^. The volume of dry air during thermal expansion can be calculated using Gay-Lussac’s Law:

4 $$\frac{{V}_{0}}{{T}_{0}}=\frac{{V}_{dry}}{T}$$

where *T* is the temperature of the chip, *T*_0_ is the initial temperature of 298.15 K (25 °C), and *V*_0_ is the initial volume. According to Eq. (4), the relative volume increase of dry air due to thermal expansion (*ΔV*_*thermal*_/*V*_0_) can be expressed as follows:

5 $$\frac{\Delta {V}_{thermal}}{{V}_{0}}=\frac{{V}_{dry}-{V}_{0}}{{V}_{0}}=\frac{T-{T}_{0}}{{T}_{0}}$$

According to Eq. (5), during the PCR amplification process, the volume expansion of dry air is shown by the gray line in Fig. 2b, and the volume of dry air only expands by a factor of 0.23.

From Eqs. (3) and (4), the relative volume increase of water vapor when the temperature increases to *T* is given by the following:

6 $$\frac{\Delta {V}_{water}}{{V}_{0}}=\frac{{p}_{WV}T}{(p-{p}_{WV}){T}_{0}}$$

Due to the combined effects of water vapor and thermal expansion, the relative maximum increase of gas volume is obtained according to Eqs. (5) and (6):

7 $$\frac{\Delta V}{{V}_{0}}=\frac{\Delta {V}_{water}}{{V}_{0}}+\frac{{V}_{thermal}}{{V}_{0}}=\frac{pT}{(p-{p}_{WV}){T}_{0}}-1$$

The maximum increase in total gas volume can be calculated based on Eq. (7), as shown by the blue line in Fig. 2b, which represents a 6.45-fold increase. This increase is significantly greater than the volume increase caused by the thermal expansion of dry air alone, which indicates that the main reason for the significant increase in bubble volume is the presence of a large amount of water vapor entering the bubbles. These calculated results align with the findings from the IEE, supporting the hypothesis that during the heat reaction process, the high saturation vapor pressure of water at elevated temperatures leads to substantial water evaporation, which is the key driving factor behind bubble formation.

### Pressurizing effect of water vapor

The pressure inside the porous PDMS was increased by water vapor, and bubbles eventually formed, which was further confirmed by a bubble formation experiment using a degassed PDMS block. The PDMS block was degassed under various low-pressure conditions while being soaked in 25 °C water until no bubbles were visible on the surface of the block. If no water vapor enters during the temperature increase and the gas volume remains constant, the air pressure inside the PDMS will increase to 1.23 times its initial pressure. Therefore, if the initial pressure of the PDMS is less than 82.378 kPa (101.325 kPa/1.23 = 82.378 kPa), the internal pressure will not exceed 101.325 kPa when the temperature is raised, so no bubbles will form due to the increase in temperature. However, when the cold water surrounding the degassed PDMS at 82.4 kPa was rapidly replaced with hot water at 95 °C and the PDMS was kept at 95 °C in a water bath heater for 30 min, bubbles still appeared on the PDMS surface. Considering the incomplete degassing of PDMS and the influence of small, invisible bubbles on its surface, the initial degassed pressure was further reduced to 70.0 kPa. Bubbles persisted, which proves that water vapor increases the pressure inside the porous PDMS. The calculation results indicate that for the same volume, internal pressures of 70.0 kPa and 82.4 kPa inside the PDMS can reach 170.964 kPa and 186.275 kPa, respectively, due to the combined influence of heating and water vapor equilibrium. Both pressures exceed 1 atm., resulting in bubbles that are 4.15 and 5.06 times the air volume for pressures of 170.964 kPa and 186.275 kPa, respectively (Fig. 2c). These bubbles may have formed despite the small amount of air that is dissolved in the preprepared 95 °C hot water because this hot water is in a state of dissolution equilibrium and therefore cannot easily provide additional air to the PDMS surface. Therefore, despite the presence of nucleation sites on the PDMS surface, the lack of air hinders the formation of more bubbles, and fewer bubbles are generated mainly from the inside of the PDMS. When the surrounding cold water was gradually heated to 95 °C, more bubbles formed on the surface of the PDMS. The cold water contains a higher concentration of dissolved air, which can be gradually released and replenished into the bubbles during the slow heating process, resulting in the formation of more air bubbles. This experiment provided additional evidence to support that water vapor was the primary cause of the bubbles.

### Release of dissolved air in water

In addition to the thermal expansion of air and the equilibrium of water vapor in PDMS, air molecules dissolved in water solutions can also escape and form bubbles as the temperature increases^35^. An experiment was conducted to measure the relative volume of overflow bubbles in water using the experimental setup shown in Fig. 2d. The glass tube was filled with water and FC-40 oil (3 M), a liquid with a higher density (1.85 g/mL) than water, and connected to an external container filled with FC-40 via a hose at the bottom of the tube. Throughout the experiment, the total amount of water in the tube remained constant, and the tube was maintained at 1 atm. After heating the glass tube in a 95 °C water bath for 30 min, ~8.0% of the total volume of water overflowed as gas at the top of the tube. When the water tube was then moved to a 25 °C water bath, the gas volume decreased to 1.2% of the water volume. The marked decrease in gas volume was due to the presence of more water vapor in the high-temperature gas, which condensed upon cooling. The released air in the bubble cannot dissolve back into the water immediately. This experiment confirmed that dissolved air in water was also a source of bubbles, but the main component of bubbles at 95 °C was water vapor.

### Process of bubble formation

Based on the experiments and analysis above, the main reasons for bubble formation in the PDMS microchips during PCR were water vapor equilibrium, air thermal expansion, and the release of dissolved air in the solution. Among these factors, water vapor had the greatest impact. The process of bubble formation can be inferred as shown in Fig. 2e. At 25 °C, a significant amount of cold air and a small amount of water vapor are present in the porous PDMS. In addition, the PCR solution contains some dissolved gas molecules. These gases all act as bubble nucleation sites. As the temperature increases, water vapor molecules enter the porous PDMS, forming small bubbles on the surface of the PDMS. Subsequently, many water vapor molecules and the air molecules released from the PCR solution enter these small bubbles, leading to the formation of larger bubbles on the PDMS surface.

Air residues may be present at defects in the chip channels and on the PDMS surface, which can also serve as bubble nucleation sites. Water vapor can enter these nucleation sites, resulting in the formation of larger bubbles. Blocking defects and surface treatments can reduce the number of nucleation sites on the PDMS surface, thus significantly reducing the total number of bubbles. However, bubbles cannot be completely eliminated because the nucleation sites within the PDMS cannot be removed.

### “Respiratory” phenomenon of PDMS during PCR

**Fig. 3:**
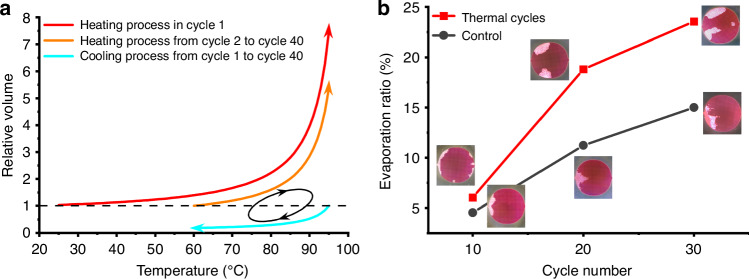
“Respiration” phenomenon of PDMS during PCR. **a** Calculations show that moist hot air overflows from the surface of PDMS during the PCR thermal cycling process, while dry cold air is drawn back into the porous PDMS during the cooling process. **b** Multiple cycles of alternating heating and cooling in the PCR process result in more liquid evaporation than thermal evaporation for the same time and temperature

Considering the evaporation of water and condensation of water vapor, the internal water in PDMS during PCR is no longer a simple diffusion process driven by concentration but rather a repeated evaporation-diffusion process driven by pressure. Dry air is continuously entering, and moist air escapes and condenses, which is referred to as a respiratory phenomenon. When the temperature drops from 95 °C to the annealing temperature of DNA at 60 °C, the saturation vapor pressure of water vapor drops to 19.932 kPa. Due to the condensation of water vapor and the cooling-induced decrease in gas volume, air from the external environment is drawn into the PDMS. During the next heating cycle, the volume expansion caused by thermal expansion and water evaporation carried a large amount of water vapor out of the PDMS (Fig. 3a). Since PCR requires multiple cycles of temperature changes, this “respiration” process accelerates the evaporation of the PCR solution.

Compared to macroscopic PDMS blocks, microfluidic chips have a larger surface area-to-volume ratio for the solution, which accelerates bubble formation. To investigate the bubble phenomenon, a PDMS cavity chip whose substrate was a silicon wafer with good thermal conductivity was used (Fig. S1). After the cavity and channels were filled with red dye, the chip was placed in an in situ PCR instrument (GeneExplorer, Bioer Tech., China) with mineral oil added between the hot plate and the chip to ensure good thermal contact. The sample inlet was sealed, while the sample outlet remained open. Within 30 s, the system temperature increased from 25 to 95 °C, and bubbles rapidly formed in the chip. After another 30 s, almost all of the dye was expelled through the outlet. This finding indicates that compared to macroscopic PDMS blocks, the speed of bubble formation in the channels and chambers of the chip is significantly faster.

Next, the “respiration” phenomenon was investigated with a PDMS cavity chip during the thermal cycling process. By sealing both the inlet and outlet, the solution cannot overflow through the outlet but can only be lost through the PDMS layer. Although the total accumulation time of heating and cooling in the thermal cycling experiment was the same as that in the control experiment (Fig. S2), the amount of solution remaining in the thermally cycled chip was less than that in the control experiment, and the difference became more pronounced as the number of cycles increased (Fig. 3b). This finding indicates that during the rapid heating and cooling of the PCR, “respiration” accelerates the evaporation of water. In addition, as the volume of bubbles in the PDMS increases, respiration-driven evaporation becomes more intense.

### Previous publication’s experimental data analysis with the water vapor enhancement theory

According to previous study^29^, the denaturation temperature for PCR was set to 93 °C (366.15 K), and the pressure used in the hydration lines was 14 pounds/square inch, which is equivalent to 96.50 kPa. According to Note S1, if only thermal expansion is considered, a pressure of 23.11 kPa would be sufficient to suppress bubble formation. However, when considering the saturation vapor pressure of water, a pressure of 97.71 kPa is required to completely prevent bubble formation. The applied pressure in this study is much greater than 23.11 kPa and close to the calculated values, effectively suppressing bubble formation, which highlights the importance of factoring in the impact of water vapor when designing chips.

In another 2021 study^36^, the PCR solution was scraped into a silicon-based nanoliter open-well array and covered with a glass coverslip. Paraffin oil and a thin layer of PDMS were placed between the array and the coverslip. Then, digital PCR (dPCR) was performed in a high-pressure microheater device at a pressure of 4 atm. The results of this experiment might be explained by the “respiration” effect. The initial pressure of the porous PDMS (1 atm.) was much lower than the pressure outside the chip (4 atm.), the top surface of the PDMS was blocked by the coverslip, outside air slowly entered the porous PDMS, and water vapor inside the chip could not flow out against the pressure. When the pressure outside the chip was reduced to 2 atm., the “respiration” effect recovered, and the water loss became severe. We also hypothesized that severe water loss would persist if the reaction time was longer, even at a pressure above 4 atm., due to the gradually decreasing pressure differential. Therefore, we proposed the use of high-pressure liquid instead of high-pressure air to isolate the chip from the air, which could prevent the “respiration” effect.

### Bubble elimination with a high-pressure liquid

To prevent bubble formation and water loss caused by the respiratory phenomenon of PDMS during thermal cycling, a high-pressure liquid seal technique was developed. The chip surface was completely and uniformly sealed by a liquid environment. By maintaining a higher pressure inside the liquid environment than that of porous PDMS, bubble formation was effectively avoided during temperature changes. In addition, the liquid-phase environment isolated the external air, thereby preventing the respiratory phenomenon during heating and cooling.

**Fig. 4:**
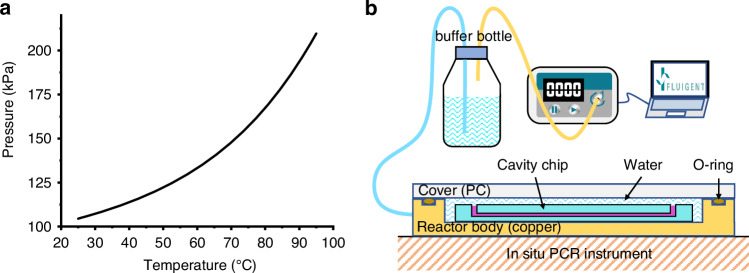
Suppression of bubble formation by a high-pressure liquid-phase environment. **a** Pressure‒temperature curves of moist air inside the porous PDMS. **b** Schematic of the high-pressure liquid-phase thermal reaction platform

When the temperature increases from 25 to 95 °C, the total pressure caused by gas expansion and water vapor ingress can reach a maximum of 209.643 kPa (Fig. 4a). Therefore, an additional pressure of at least 108.318 kPa (209.643−101.325 kPa) can prevent the gas within the PDMS from escaping and forming bubbles. Moreover, a previous publication showed that an additional pressure of at least 101.325 kPa is sufficient to prevent the formation of such bubbles^37^, further supporting that this additional pressure can also prevent the dissolved gas in the PCR solution from escaping and forming bubbles.

This method was also highly efficient for eliminating very small bubbles in microfluidic channels or invisible “bubble nucleation sites” at the liquid/PDMS interface. The high-pressure environment reduced the volume of these bubbles or drove the gases into the interior of the PDMS. Bubble formation was completely prevented by simply increasing the pressure of the liquid environment. Furthermore, during the heating process, the water phase environment outside the PDMS facilitated the entry of water vapor into the PCR, reducing the loss of PCR solution to a negligible extent.

To validate our calculations, we designed and fabricated a pressure-liquid reactor (Fig. 4b). To minimize heating and cooling times, highly heat conductive copper manufactured by computer numerical control was used as the reactor body. The cover of the reactor was made of transparent PC, and the sidewalls of the reactor body and the cover were sealed with flexible O-rings. In addition, the sidewalls of the reactor were connected to a buffer bottle and a pressure pump through tubes to control the pressure inside the reactor.

A PDMS cavity chip filled with red dye was placed inside the reactor filled with water. Then, the reactor was sealed using a PC cover, and no residual air remained in the reactor. The pressure of the reactor was increased by 109 kPa. After the thermal cycles of PCR, no significant changes were observed in the cavity chip, indicating that the high-pressure liquid-phase heating environment can effectively suppress bubble formation and solution evaporation.

### Performing dPCR with a high-pressure liquid

**Fig. 5:**
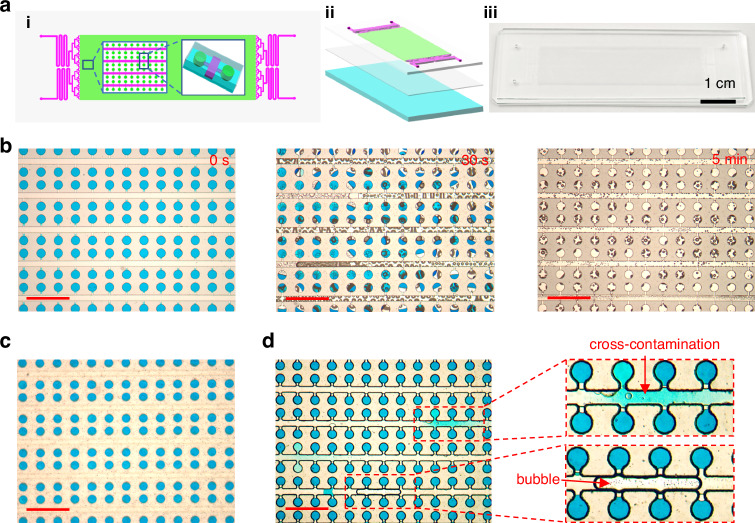
Bubble prevention by a high-pressure liquid. **a** dPCR chip. (i) Schematic of the device, with insets showing the array and chamber geometries. (ii) Schematic of the layered device structure, from top to bottom, the reaction layer, the blank layer, and the glass substrate. (iii) Photograph of the chip. **b** Micrographs of the PDMS chip during PCR in air. **c** Micrograph of the chip in the high-pressure liquid reactor. **d** Micrograph of the chip in a liquid reactor without high pressure. Scale bars, 500 μm (**b**–**d**)

Next, the inhibitory effect of high pressure on solution evaporation and bubble formation in dPCR chips was investigated. The dPCR chip made of PDMS, consisting of a PDMS structural layer, a PDMS blank layer and a glass substrate, is shown in Fig. 5a. The structural layer consists of microchannels and an array of microreaction units. The chip contains 15,360 individual cylindrical microchambers (height = 60 μm, diameter = 100 μm). The rows of the chambers are connected to the main channels (height = 60 μm, width = 60 μm) through branch channels (height = 10 μm, width = 20 μm). The volume of each chamber was 0.47 nL. A chip filled with blue dye and isolated with oil (Fig. S3) was directly heated on the hot plate of the in situ PCR instrument. As shown in Fig. 5b, numerous bubbles were generated inside the chip during the thermal cycling process, and the rapid expansion of these bubbles extruded the solution from the chip, accelerating the loss of the solution. After only 5 min, almost all of the solution inside the chip was lost. However, when the chip filled with blue dye was placed into the pressure-liquid reactor and PCR was conducted under a high-pressure water environment, no bubbles were observed inside the chip, and the amount of solution did not decrease (Fig. 5c). When the chip was placed in a water environment without high pressure, a significant number of air bubbles and cross-contamination between the reaction units were observed during PCR (Fig. 5d). The high-pressure liquid sealing method eliminates the need for various valve structures commonly used in PDMS chips to prevent bubble formation and the need for sealing covers to prevent evaporation.

**Fig. 6:**
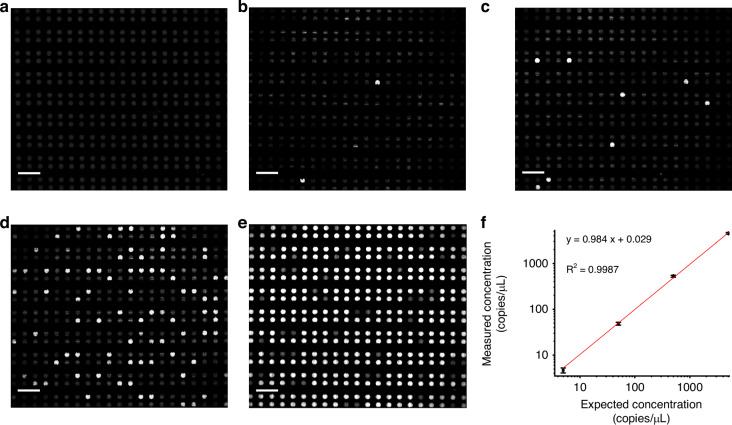
Results of dPCR performed on chips with different dilutions of the target DNA template. Fluorescence images of negative (**a**), 5 copies/μL (**b**), 5 × 10 copies/μL (**c**), 5 × 10^2^ copies/μL (**d**), and 5 × 10^3^ copies/μL (**e**), respectively. **f** Linear relationship between the measured concentration in the dPCR chip and the expected concentration. Scale bars, 400 μm (**a**–**e**)

Finally, a high-pressure liquid-phase thermal cycling method was used to detect the synthesized fragment of the SARS-CoV-2 nucleocapsid protein-encoding gene. The sample was serially diluted tenfold to concentrations ranging from 5 copies/μL to 5 × 10^3^ copies/μL (averages of 2.35 × 10^−3^ copies to 2.35 copies per 0.47 nL chamber). The detection results exhibited a high degree of linearity (R^2^ = 0.9987) (Fig. 6, Table S1). This result demonstrated that applying pressure can ensure the integrity and stability of the reaction system in the chip and can completely prevent bubble formation in the PDMS chip. However, fluorescent signals also appeared in the microfluidic channel of the chip in a liquid environment without high pressure after thermal cycling (Fig. S4).

A high heat capacity of 4.2 kJ/(kg·K) and a low thermal conductivity of 0.6 W/(m·K) will increase the thermal load on the system and increase the thermal cycling time. Therefore, minimizing the volume of water is essential. Fortunately, the volume of water is relatively small because the chip is thin. Furthermore, employing dynamic circulation of water at different temperatures to drive the thermal cycling of the chip can further accelerate PCR. Hideyuki Terazono et al. proposed a water-circulation-driven heating method based on a water cycle that can perform 40 thermal cycles (1 s denaturation and 3 s extension) in 3.5 min, which is an order of magnitude faster than conventional rapid PCR systems^38^. Thus, applying high pressure to the water circulation system not only solves the problems of bubble formation and solution evaporation but also shortens the time of thermal cycling and even provides faster heat exchange than conventional semiconductor heating.

## Conclusion

The present study highlights the significance of water vapor as a critical factor in the design and manufacture of PCR chips. The formation of bubbles in the PCR chips was primarily attributed to the excessive saturation vapor pressure of water at high temperatures. Even intact PDMS blocks could generate bubbles in solution at the same temperature. Under high-temperature PCR conditions, the amount of water vapor inside the bubbles far exceeded that of dry air. Theoretical analysis revealed that at 1 atm., the thermal expansion of air from 25 to 95 °C resulted in only a 0.23-fold increase in volume, whereas heating resulted in a 6.45-fold increase in water vapor volume. Considering the rapid evaporation of water during chip heating and the rapid condensation of water vapor during cooling, significant pressure differences occurred within the PDMS, resulting in the expulsion and inhalation of a large amount of air, which is referred to as the respiratory phenomenon. This phenomenon accelerated the evaporation of the PCR solution. When the absolute ambient pressure around the PCR chip exceeded 209 kPa, no bubbles escaped from the solution. Furthermore, this pressure could also suppress the outgassing of air dissolved in the liquid at high temperatures. Based on the calculations, we propose the use of a high-pressure water seal method, which employs high pressure to prevent gas exhalation and a water environment to prevent gas inhalation, effectively eliminating both the occurrence of bubbles and the respiratory phenomenon. This method only modifies the heating environment of the chip, freeing the chip from the constraints of valves or seals, significantly reducing the complexity and cost of manufacturing disposable PCR chips, and offering vast application prospects.

## Materials and methods

### IEE with PDMS blocks of different thicknesses

First, PDMS blocks of different thicknesses were prepared. PDMS prepolymer and curing agent (Sylgard 184, Dow Corning) were mixed at a 10:1 ratio by mass. The PDMS mixture was degassed using a vacuum device to remove bubbles and poured onto a silicon wafer. The thickness of the PDMS was determined by the mass of the PDMS mixture. The wafer covered with the PDMS mixture was then placed on a horizontal platform for 2 h to allow the mixture to level out. Next, the wafer was heated on an 80 °C hot plate for 30 min. After curing, the PDMS was peeled off from the silicon wafer and cut into square blocks measuring 2 cm × 2 cm. Finally, the PDMS blocks were bonded to glass slides after plasma treatment. Prior to the isothermal expansion experiment, the PDMS blocks bonded to the glass slides were heated in an oven overnight to reach the desired experimental temperature. A quartz glass square cup containing cold water was heated to the desired temperature using a water bath. Bubbles on the glass walls were removed using an overhead stirrer. The preheated PDMS blocks were then quickly placed into the cup. The PDMS blocks were photographed for analysis.

### Manufacture of both master mold and chips

The soft lithography technique was employed to fabricate the PDMS chips. The mold of the cavity chip was fabricated with one layer of SU-8 photoresist (Microchem). A 60-μm thick layer of SU-8 3050 was spin-coated onto the silicon wafer with a self-levelling time of 2 h. After soft baking, exposure, development, postexposure baking, and hard baking at 170 °C for 30 min, the cavity mold was prepared.

The cavity mold was treated with trichloro(1H,1H,2H,2H-perfluorooctyl) silane (Sigma-Aldrich) to prevent PDMS adhesion. The PDMS mixture (10:1) poured onto the cavity mold was heated at 80 °C for 30 min. The cured PDMS was then peeled off from the mold and cut into individual cavity layers. The inlet and outlet were punched in the cavity layer. The cavity layer and the silicon substrate were plasma treated separately for 60 s (150 W) and then bonded together. The cavity chip was fabricated after baking at 80 °C for 2 h to increase the bond strength.

The mold of the dPCR chip consisted of two layers of SU-8 photoresist. First, a 10 μm layer of SU-8 3005 was spin-coated on a silicon wafer and patterned on the silicon wafer using a method similar to that used for cavity mold fabrication. Then, a 60 μm layer of SU-8 3050 was patterned.

The PDMS mixture (10:1) was cured on a dPCR mold and then cut and punched to prepare the reaction layer. The PDMS blank layer was prepared by spin-coating a PDMS mixture onto a glass slide at 3000 rpm for 1 min and curing at 80 °C for 15 min. The reaction layer and the blank layer were then plasma treated for 60 s, bonded together, and baked at 80 °C for 2 h.

### PCR amplification in a high-pressure liquid phase environment

The forward primer (5′-GGGGAACTTCTCCTGCTAGAAT-3′), reverse primer (5′-CAGACATTTTGCTCTCAAGCTG-3′), CY5-labeled hydrolysis probe (5′-CY5-TTGCTGCTGCTTGACAGATT-MGB-3′) and template (5′-AGTAGGGGAACTTCTCCTGCTAGAATGGCTGGCAATGGCGGTGATGCTGCTCTTGCTTTGCTGCTGCTTGACAGATTGAACCAGCTTGAGAGCAAAATGTCTGGTAAAG-3′) were synthesized by Sango Biotech Co., Ltd. A 99 bp product could be amplified and traced with the probe after PCR thermal cycling. To assess the performance of the proposed dPCR chip, the template DNA was serially diluted 10-fold from 5 to 5 × 10^3^ copies/µL using nuclease-free water. The primers and probes were diluted to a concentration of 10 μM using a sterile 10 mM Tris-HCl solution (pH 7.5, sterile dilution 100 times). Each 100 µL PCR mixture consisted of 50 µL of rapid PCR master mix with 2X concentration, 10 µL of forward/reverse primers with 10 mol/L, 3 µL of CY3/CY5 fluorescent probes with 10 mol/L, 14 µL of template DNA, and 10 µL of nuclease-free water. All reagents were purchased from Sango Biotech Co., Ltd., and stored at −20 °C before use. The amplification cycle was initiated after fully denaturing the template DNA by preheating it at 95 °C for 5 min. Then, forty cycles of 95 °C for 60 s and 60 °C for 60 s were performed to amplify the target DNA.

### Image acquisition and analysis

After PCR amplification, fluorescence images of the reaction units were collected by a steroidal fluorescence microscope (Olympus, Japan). The fluorescence of probe CY5 was excited by red light at a wavelength of 650 nm, and the emission light was captured by a CCD camera (Andor, Northern Ireland) with a large field of view and high sensitivity. The fluorescence intensity of the reaction units was measured using the “Analyze Particles” module in ImageJ, which is an open-source image analysis software. Origin 2023 data processing software was used to plot the fluorescence intensity distribution of all reaction units, and the number of positive reaction units was counted.

In dPCR, the DNA template dispersed in the reaction unit obeys a Poisson distribution. The initial concentration of the DNA template in the sample detected by dPCR can be calculated by

8 $$C=-\frac{{\mathrm{ln}}(1-\frac{m}{n})}{V}$$

where *m* is the number of positive units, *n* is the total number of reaction units on the chip, and *V* is the volume of each reaction unit. The dPCR experiments were repeated three times for each concentration of template DNA to ensure the robustness and reproducibility of the assay results. The standard deviation formula was used to calculate the error of the three experiments.

## Supplementary information

Supplemental Material
