# Supplementary material for: Overcoming bubble formation in polydimethylsiloxane-made PCR chips: mechanism and elimination with a high-pressure liquid seal
Source: Microsyst Nanoeng. 2024 Sep 27;10:136. doi: 10.1038/s41378-024-00725-1 (PMC11427668; doi:10.1038/s41378-024-00725-1)
Supplement: Supplementary file 1 — Supplemental Material [file 41378_2024_725_MOESM1_ESM.docx]

Overcoming bubble formation and water loss in PDMS-made PCR chips: mechanism and elimination with high-pressure liquid seal

Shiyuan Gao^1,2,3,^ Tiegang Xu^1,2,*^, Lei Wu^1,2,*^, Xiaoyue Zhu^4^, Xuefeng Wang^1,2^, Xiaohong Jian^5^ and Xinxin Li^1,2,3,*^

^1^State Key Laboratory of Transducer Technology, Shanghai Institute of Microsystem and Information Technology, Chinese Academy of Sciences, Shanghai 200050, China

^2^College of Materials Science and Opto-Electronic Technology, University of Chinese Academy of Sciences, Beijing 100049, China

^3^School of Information Science and Technology, ShanghaiTech University, Shanghai 201210, China

^4^Metabolomics Center, Haixia Institute of Science and Technology, School of Future Technology, Fujian Agriculture and Forestry University, Fuzhou 350002 China

^5^School of Biological Engineering, Sichuan University of Science and Engineering, Yibin 644000, China

*Email: xutiegang@mail.sim.ac.cn (T.X.); wulei@mail.sim.ac.cn (L.W.); xxli@mail.sim.ac.cn (X.L.);

**
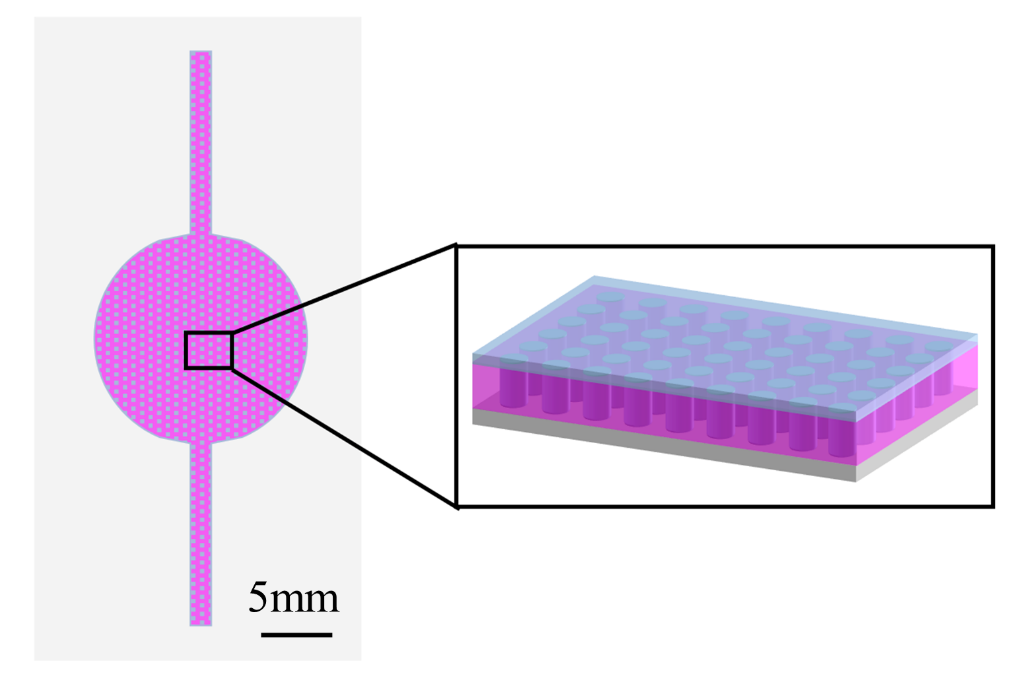
**

**Fig. S1: Schematic diagram of a PDMS cavity chip.** The cavity is 1.5 cm in diameter and 60 μm in height. The thickness of the cavity layer is 800 μm. A support pillar array with 100 μm pillar diameter and 100 μm spacing was distributed inside the cavity to prevent from the cavity collapsing.

| **10 cycles** | **** |
| --- | --- |
| **20 cycles** | **** |
| **30 cycles** | **** |

**Fig. S2:** **The temperature curve for the PDMS “respiratory” effect.** In the multi-cycle heating mode (red line), each cycle lasts for 36 seconds. The total time for temperature heating and cooling in the thermal cycle mode is equivalent to that of the control experiment (black line).

**
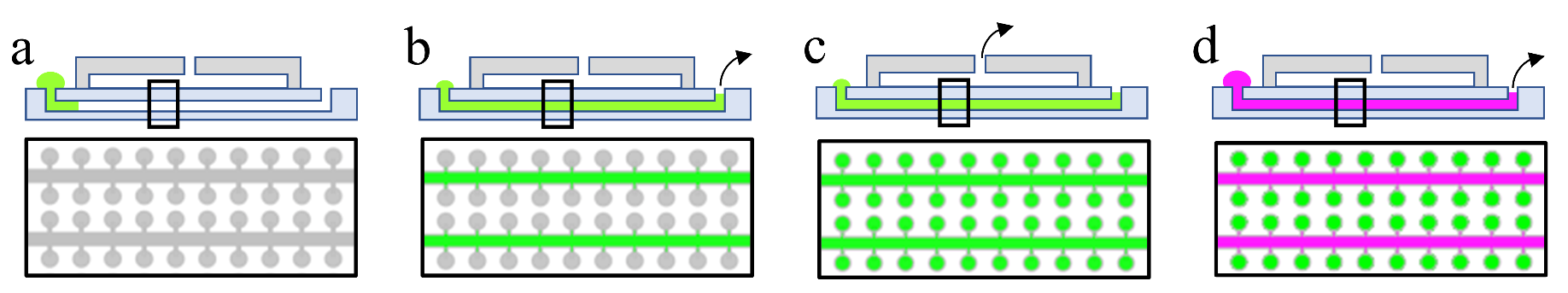
**

**Fig. S3: Sample loading and digitalization process.** (a) An auxiliary loader was placed on the surface of the chip, and the PCR solution was added to the inlet. (b) Negative pressure was applied at the outlet, allowing the PCR solution to be completely filled in the main channel inside the chip. (c) Negative pressure was applied inside the auxiliary loader, ensuring that the PCR solution fills the reaction units without any dead ends. (d) FC-40 added to the inlet was used to displace the PCR solution within the main channel and isolate the reaction units from each other.


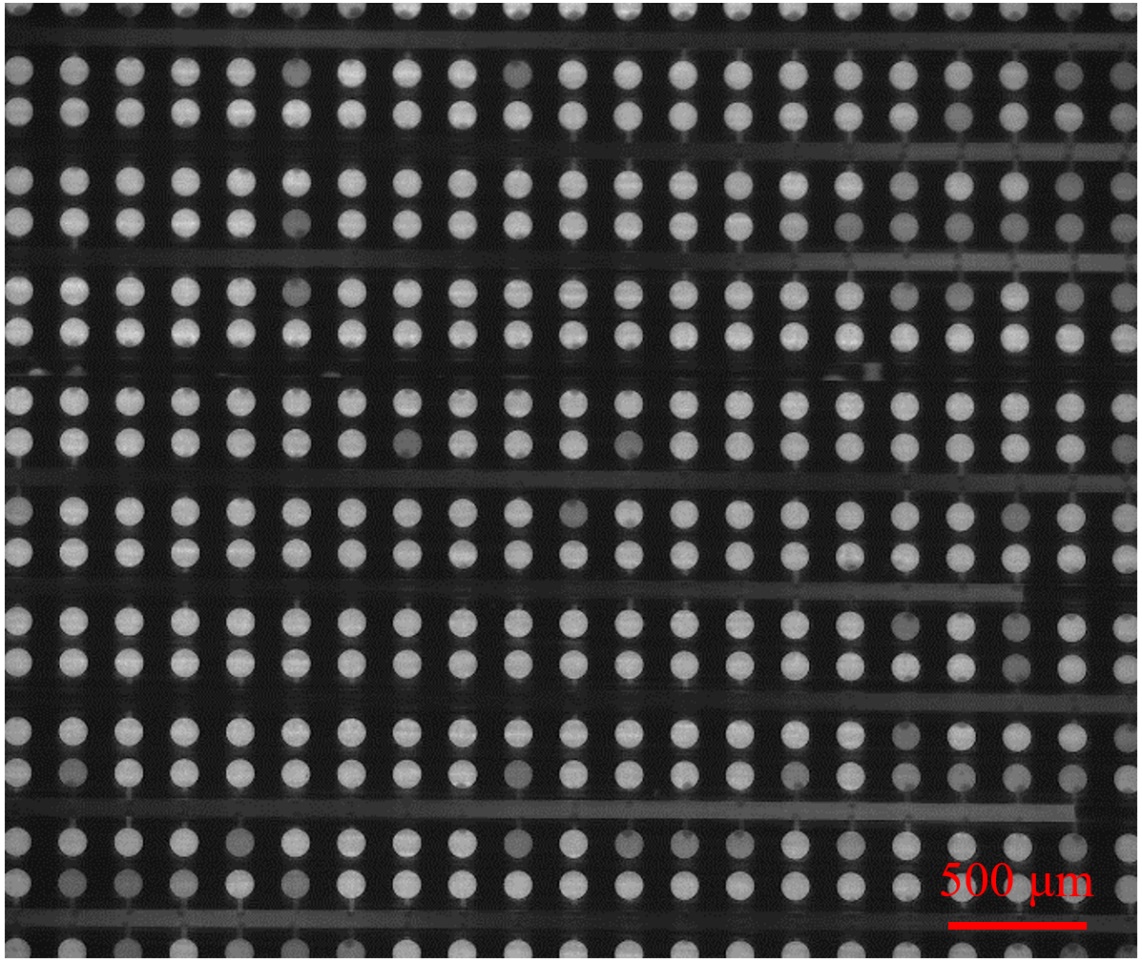


**Fig. S4: The result of dPCR performed on the chip in water environment without high-pressure.** The fluorescence signals within the microfluidic channels of the chip indicates that cross-contamination between reaction units has occurred.

**Note S1: Calculation of pressure inside the bubble**

In the process of heating a chip from an initial pressure of 101.325 kPa at room temperature of 25℃ (298.15 K) to 93℃ (366.15 K), the internal humidity of the chip can range from 0% to 100%. Based on these values, we can calculate the maximum pressure range inside the chip.

At 25℃ and 0% humidity, the air partial pressure is 101.325 kPa. When heated at constant pressure to 93℃, the air partial pressure can reach 124.435 kPa (101.325 kPa × 366.15 K/298.15 K). The temperature increase causes a pressure increase of 23.110 kPa (124.435 kPa - 101.325 kPa). At 93℃, the saturated vapor pressure of water is 78.494 kPa. At 100% humidity, the total pressure inside the chip will be 202.929 kPa (124.435 kPa + 78.494 kPa), resulting in a pressure increase of 101.604 kPa (202.929 kPa - 101.325 kPa).

At 25℃ and 100% humidity, the saturated vapor pressure of water is 3.169 kPa, and the air partial pressure is 98.156 kPa (101.325 kPa - 3.169 kPa). When heated at constant pressure to 93℃, the air partial pressure can reach 120.543 kPa (98.156 kPa × 366.15 K/298.15 K). At 100% humidity, the total pressure inside the chip will be 199.037 kPa (120.543 kPa + 78.494 kPa), resulting in a pressure increase of 97.712 kPa (199.037 kPa - 101.325 kPa).

Therefore, based on calculations, the internal pressure of the chip can increase by a maximum of 97.712 kPa to 101.604 kPa when heating from room temperature of 25℃ to 93℃.

**Table S1: The statistical analysis result of the digital PCR**

| Expected concentration (copies/μL) | Number of positive units | Measured concentration (copies/μL) |
| --- | --- | --- |
| 5 | 37 | 4.668661±0.577936 |
|  | 29 |  |
|  | 35 |  |
| 50 | 321 | 48.43054±3.54378 |
|  | 345 |  |
|  | 371 |  |
| 500 | 3368 | 524.9294±26.35699 |
|  | 3501 |  |
|  | 3204 |  |
| 5000 | 13653 | 4498.636±188.5739 |
|  | 13526 |  |
|  | 13324 |  |
